# Trends in publication of evidence-based Traditional Iranian medicine in endocrinology and metabolic disorders

**DOI:** 10.1186/2251-6581-12-49

**Published:** 2013-12-19

**Authors:** Shirin Hasani-Ranjbar, Hoda Sadat Zahedi, Mohammad Abdollahi, Bagher Larijani

**Affiliations:** 1 Obesity and Eating Habits Research Center, Endocrinology and Metabolism Molecular-Cellular Sciences Institute, Tehran University of Medical Sciences, Tehran, Iran; 2 Endocrinology and Metabolism Research Center, Endocrinology and Metabolism Clinical Sciences Institute, Tehran University of Medical Sciences, Tehran, Iran; 3 Faculty of Pharmacy, Pharmaceutical Sciences Research Center, Tehran University of Medical Sciences, Tehran, Iran

**Keywords:** Complementary medicine, Alternative medicine, Herbal medicine, Traditional Iranian medicine, Pharmacologic treatments, Pharmaceutical companies

## Abstract

Traditional Iranian medicine (TIM) is a main part of complementary and alternative medicine (CAM). The popularity and use of alternative therapies are increasing due to adverse effects and ineffectiveness of pharmacologic treatments in some cases. Herbal medicine is one of the methods of traditional therapy that plays a key role in the treatment of various diseases specifically in diabetes mellitus, hyperlipidemia and obesity that are growing rapidly in the world. In this article, trends of scientific publications of Iranian medicine in endocrinology and metabolic disorders have been investigated. Our data show that the numbers of related researches have uptrend from 2000 till now. These data are valuable to pharmaceutical companies to get the idea to invest and produce effective drugs.

**Electronic supplementary material:**

The online version of this article (doi:10.1186/2251-6581-12-49) contains supplementary material, which is available to authorized users.

## Background

Obesity, diabetes mellitus and metabolic disorders are the most major health problems with increasing prevalence all over the world [1, 2]. For example, the global prevalence of type 2 diabetes for all age-group in the world was about 2.8% in the year 2000 and it is appraised to become 4.4% by 2030 [3]. The main reasons of these disorders are change in behaviors, nutrition and sedentary lifestyle [4]. In addition, estimated total numbers of obese and overweight adults in the world in 2005 were respectively 369 million and 937 million [5]. In comparison to 20 years ago, these figures have been doubled [6]. It is estimated that these numbers will be 537 million and 1.35 billion for obese and overweight adults, respectively [5]. The reported prevalence of obesity and overweight in Iran was 42.8% in men and 57% in women in 2005 [7]. The numbers are estimated to be 54% and 74%, respectively in 2015 [8].

The popularity and use of alternative therapies are increasing dramatically because pharmacologic treatments have adverse effects and are somehow ineffective in some conditions. In addition, alternative medicine appears to be more conformable with patients’ beliefs and values [9]. Herbal medicine is one of the methods used in traditional medicine that is the most popular complementary and alternative medicine (CAM) modality and plays a key role in treatment of several disorders specifically in Eastern countries and some developed countries like Germany, France, Italy and United states [10–13]. During recent decades, modern medicine has achieved explosive developments, but plants are still a cornerstone of health care and medical prescriptions [13]. Based on the World Health Organization (WHO), 65-80% of the world’s population living in developing countries need to herbal medicines because they have no access to modern medicine [14] due to poverty and lack of safe modern drugs. Evaluation of medicinal plants efficacy for treatment of some disorders such as diabetes has been recommended by WHO [15].

Nowadays, herbal products are used for prevention, mitigation and treatment of some diseases like a drug but some of them has been found unsafe and only a few have been evaluated adequately by modern tests [13, 16].

Considering above points, it is clear that many traditional plants are used for treatment of diseases in Iran and throughout the world as adjuncts to conventional therapy. In this paper, we have introduced the studies which are about herbal medicines performed in Iran. Our investigation has been conducted based on area of studies including diabetes mellitus, hyperlipidemia, obesity and hyperprolactinemia, phytochemistry and pharmacologic studies. Moreover, highly cited articles in each category have been presented. Data from this paper will be valuable not only for identifying the trends in evidence based traditional medicine in Iran, but also for researchers to applying highly cited studies.

To find useful medicinal plants in endocrinology and metabolic disorders,  PubMed, Scopus and Google scholar were searched up to now. The search terms were “plant”, “herb”, “traditional”, “herbal medicine”, “naturopathy”, “phytotherapy” or “healing plant” and “Iran”. The specific search terms for diabetes, hyperlipidemia, obesity and hyperprolactinemia were “diabetes”, “hyperlipidemia” or “dyslipidemias”, “obesity” or “obese” or “overweight”, “hyperprolactinemia” or “prolactinoma” or “prolactin” respectively.

### Area of studies

#### Diabetes

The number of published studies regarding the effects of herbal medicine on diabetes is high. Forty four articles were found in this field [10, 17–59]. Highly cited articles in these areas were: “The efficacy of Silybummarianum (L.) Gaertn. (silymarin) in the treatment of type II diabetes: a randomized, double-blind, placebo-controlled, clinical trial” [34], which has been cited 103 times in Google scholar and 58 times in Scopus; “A systematic review of the potential herbal sources of future drugs effective in oxidant-related diseases” [55], 100 times in Google scholar and 87 times in Scopus; “Psyllium decreased serum glucose and glycosylated hemoglobin significantly in diabetic outpatients” [54], 68 times in Google scholar and 47 times in Scopus.

#### Hyperlipidemia

Fifteen studies were performed about this issue in Iran [60–74] and highly cited articles were: “Antihypertensive and antihyperlipidemic effects of Achilleawilhelmsii” [61], that cited 63 times in Google scholar and 30 times in Scopus; “Effects of anethumgraveolens and garlic on lipid profile in hyperlipidemic patients” [67], cited 46 and 21 times in Google scholar and Scopus, respectively; “The efficacy and safety of herbal medicines used in the treatment of hyperlipidemia; a systematic review” [65], cited 28 times in Google scholar and 22 times in Scopus.

#### Obesity

In this field, 12 related articles have been published in Iran [75–87]. Highly cited article is: “A systematic review of the efficacy and safety of herbal medicines used in the treatment of obesity” [79], which cited 93 and 65 times in Google scholar and Scopus, respectively.

#### Hyperprolactinemia

The search results showed that 2 articles have been published about this issue up to now in Iran [88, 89] including: “A systematic review on the efficacy of herbal medicines in the management of human drug-induced hyperprolactinemia: Potential sources for the development of novel drugs” [89], cited 11 and 10 times in Google scholar and Scopus, respectively and “Effect of Vitexagnus - Castus L. leaf and fruit flavonoidal extracts on serum prolactin concentration” [88], cited 4 times in Google scholar and 3 times in Scopus.

#### Phytochemistry and pharmacological studies

In the results of our search, there were 11 studies that had investigated the phytochemistry and pharmacological properties of a plant or plants of a specific region in Iran [90–100]. The highly cited published articles about these topics are: “Traditional uses, phytochemistry and pharmacology of asafoetida (Ferula assa-foetida oleo-gum-resin)-a review” [92], which has been cited 22 times in Google scholar and 17 times in Scopus and “Ethnobotanical survey of herbal remedies traditionally used in Kohghiluyehva Boyer Ahmad province of Iran” [96], that cited 13 and 5 times in Google scholar and Scopus.

### Trends

**Figure 1:**
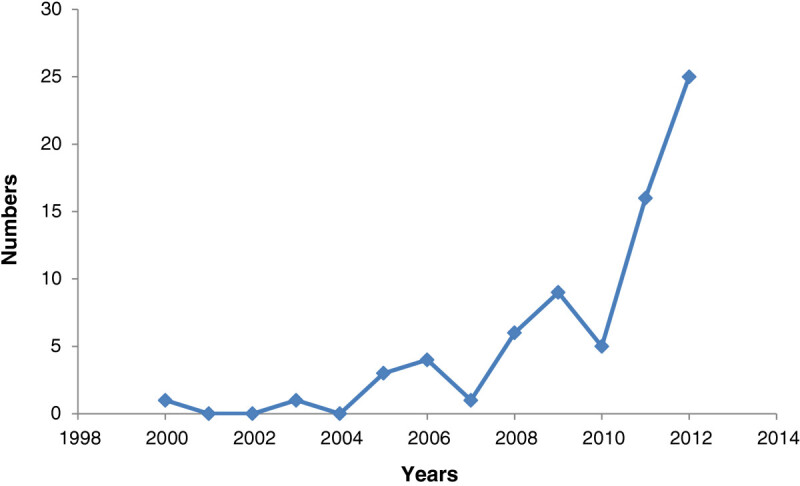
**Numbers of herbal medicine published in Iran from 2000 to 2013.**

According to the area of studies, highly cited studies have been shown in Table 1. In addition, Figure 1 illustrates the trend of published articles in the recent years. Trends in these articles show that the number of evidence-based studies about traditional medicine is growing in Iran.

**Table 1:** **Highly cited articles according to the area of studies**

Category of articles	Author	Year	Number of citation
Diabetes	FallahHuseini et al.	2006	103
	Hasani-Ranjbar et al.	2009	100
	Ziai et al.	2005	68
Hyperlipidemia	Asgary et al.	2000	63
	Kojuri et al.	2007	46
	Hasani-Ranjbar et al.	2010	28
Obesity	Hasani-Ranjbar et al.	2009	93
Phytochemistry and pharmacologic studies	Iranshahi et al.	2011	22
	Mosaddegh etal	2012	13

## Conclusion

The TIM has been under attention of the researchers in the recent years. The number of related researches has uptrend from 2000 until now. Our data show a great enthusiasm towards the TIM specifically herbal medicine that has a historical background in Iran. This paper opens a new window towards future studies.

## Authors’ original submitted files for images

Below are the links to the authors’ original submitted files for images. Authors’ original file for figure 1

## References

[1] Consultation W (2000). Obesity: preventing and managing the global epidemic.

[2] Atalay M, Laaksonen DE (2002). Diabetes, oxidative stress and physical exercise. Journal of Sports Science and Medicine.

[3] Wild S, Roglic G, Green A, Sicree R, King H (2004). Global prevalence of diabetes estimates for the year 2000 and projections for 2030. Diabetes care.

[4] Hardeman W, Griffin S, Johnston M, Kinmonth A, Wareham N (2000). Interventions to prevent weight gain: a systematic review of psychological models and behaviour change methods. International journal of obesity and related metabolic disorders: journal of the International Association for the Study of Obesity.

[5] Kelly T, Yang W, Chen C, Reynolds K, He J (2008). Global burden of obesity in 2005 and projections to 2030. International journal of obesity.

[6] James PT, Rigby N, Leach R (2004). The obesity epidemic, metabolic syndrome and future prevention strategies. European Journal of Cardiovascular Prevention & Rehabilitation.

[7] Janghorbani M, Amini M, Willett WC, Gouya MM, Delavari A, Alikhani S (2007). First nationwide survey of prevalence of overweight, underweight, and abdominal obesity in Iranian adults. Obesity.

[8] WHO (2008). Chronic diseases are the major cause of death and disability worldwide.

[9] Astin JA (1998). Why patients use alternative medicine. JAMA: the journal of the American Medical Association.

[10] Dabaghian FH, Kamalinejad M, Shojaei A, Fard MA (2012). Presenting anti-diabetic plants in Iranian traditional medicine. Journal of Diabetes and Endocrinology Vol.

[11] Hasani-Ranjbar S, Larijani B, Abdollahi M (2008). A systematic review of Iranian medicinal plants useful in diabetes mellitus. Arch Med Sci.

[12] Sadighi J, Maftoun F, Moshrefi M (2004). Complementary and alternative medicine (cam): knowledge, attitude and practice in Tehran.

[13] Calixto J (2000). Efficacy, safety, quality control, marketing and regulatory guidelines for herbal medicines (phytotherapeutic agents). Braz J Med Biol Res.

[14] Akerele O (1992). WHO guidelines for the assessment of herbal medicines. Fitoterapia.

[15] Kim J-D, Kang S-M, Park M-Y, Jung T-Y, Choi H-Y, Ku S-K (2007). Ameliorative anti-diabetic activity of dangnyosoko, a Chinese herbal medicine, in diabetic rats. Bioscience, biotechnology, and biochemistry.

[16] Klepser TB, Klepser ME (1999). Unsafe and potentially safe herbal therapies. Am J Health Syst Pharm.

[17] Abdollahi M, Tabatabaei-Malazy O, Larijani B (2012). A systematic review of in vitro studies conducted on effect of herbal products on secretion of insulin from Langerhans islets. J Pharm Pharm Sci.

[18] Bahadoran Z, Mirmiran P, Hosseinpanah F, Hedayati M, Hosseinpour-Niazi S, Azizi F (2011). Broccoli sprouts reduce oxidative stress in type 2 diabetes: a randomized double-blind clinical trial. Eur J Clin Nutr.

[19] Bahadoran Z, Mirmiran P, Hosseinpanah F, Rajab A, Asghari G, Azizi F (2012). Broccoli sprouts powder could improve serum triglyceride and oxidized LDL/LDL-cholesterol ratio in type 2 diabetic patients: a randomized double-blind placebo-controlled clinical trial. Diabetes Res Clin Pract.

[20] Bakhtiuary Z (2011). Herbal medicines in diabetes. Iranian Journal of diabetes and obesity.

[21] Falah Hosseini H, Ardeshir Larijani Mohammad B, Fakhrzadeh H, Darvish ZF, Rahmani M, Heshmat R (2006). The clinical investigation of citrullus colocynthis (l.) schrad. fruit in treatment of type ii diabetic patients; a randomized, doubleblind, placebo-controlled study. Journal of Medicinal Plants.

[22] Huseini HF, Darvishzadeh F, Heshmat R, Jafariazar Z, Raza M, Larijani B (2009). The clinical investigation of Citrullus colocynthis (L.) schrad fruit in treatment of Type II diabetic patients: a randomized, double blind, placebo-controlled clinical trial. Phytother Res.

[23] Falah Hosseini H, Fakhrzadeh H, Ardeshir Larijani Mohammad B, Shikh Samani A (2006). Review of anti-diabetic medicinal plant used in traditional medicine. Journal of Medicinal Plants.

[24] Fallah Huseini H, Heshmat R, Mohseni F, Jamshidi AH, Alavi SHR, Ahvasi M (2008). The efficacy of rheum ribes L. stalk extract on lipid profile in hypercholesterolemic type II diabetic patients: a randomized, double-blind, placebo - controlled, clinical trial. Journal of Medicinal Plants.

[25] Fallah Huseini H, Hooseini P, Heshmat R, Yazdani D, Hemati Moqadam HR, Rahmani M (2006). The clinical investigation of Securigera securidaca (L.) (Degen & Doerfler) seeds in type II diabetic patients; a randomized, double-blind, placebo-controlled study. Journal of Medicinal Plants.

[26] Fallah Huseini H, Kianbakht S, Heshmat R (2012). Cynara scolymus L. In treatment of hypercholesterolemic type 2 diabetic patients: a randomized double-blind placebo-controlled clinical trial. Journal of Medicinal Plants.

[27] Ghorbani A (2013). Phytotherapy for diabetic dyslipidemia: evidence from clinical trials. Clinical Lipidology.

[28] Hasani-Ranjbar S, Larijani B, Abdollah M (2008). A systematic review of iranian medicinal plants useful in diabetes mellitus. Archives of Medical Science.

[29] Hashem Dabaghian F, Kamalinejad M, Shojaii A, Abdollahi Fard M, Ghushegir SA (2012). Review of antidiabetic plants in Iranian traditional medicine and their efficacy. Journal of Medicinal Plants.

[30] Heidarzadeh S, Farzanegi P, Azarbayjani MA, Daliri R (2009). Purslane Effect on GLP-1 and GLP-1 receptor in type 2 diabetes. Electronic Physician.

[31] Hosseini S, Fallah-Huseini H, Watson R, Preedy V (2008). The efficacy of Citrullus colocynthis fruit and Silybum marianum (Silymarin) in treatment of diabetes. Botanical medicine in clinical practice.

[32] Hosseyni ES, Kashani HH, Asadi MH (2012). Mode of action of medicinal plants on diabetic disorders. Life Science Journal.

[33] Huseini HF, Kianbakht S, Hajiaghaee R, Dabaghian FH (2012). Anti-hyperglycemic and anti-hypercholesterolemic effects of Aloe vera leaf gel in hyperlipidemic type 2 diabetic patients: a randomized double-blind placebo-controlled clinical trial. Planta Med.

[34] Huseini HF, Larijani B, Heshmat R, Fakhrzadeh H, Radjabipour B, Toliat T (2006). The efficacy of Silybum marianum (L.) Gaertn. (silymarin) in the treatment of type II diabetes: a randomized, double-blind, placebo-controlled, clinical trial. Phytother Res.

[35] Kamali SH, Khalaj AR, Hasani-Ranjbar S, Esfehani MM, Kamalinejad M, Soheil O (2012). Efficacy of ‘;Itrifal Saghir’ , a combination of three medicinal plants in the treatment of obesity; A randomized controlled trial. DARU, Journal of Pharmaceutical Sciences.

[36] Kassaian N, Azadbakht L, Forghani B, Amini M (2009). Effect of fenugreek seeds on blood glucose and lipid profiles in type 2 diabetic patients. Int J Vitam Nutr Res.

[37] Khadem Haghighian H, Farsad Naimi A, Pourghassem Gargari B, Ali-Asgharzadeh A, Nemati A (2011). Effect of cinnamon supplementation on blood glucose and lipid levels in type2 diabetic patients. Journal of Paramedical Sciences.

[38] Khajehdehi P, Pakfetrat M, Javidnia K, Azad F, Malekmakan L, Nasab MH (2011). Oral supplementation of turmeric attenuates proteinuria, transforming growth factor-beta and interleukin-8 levels in patients with overt type 2 diabetic nephropathy: a randomized, double-blind and placebo-controlled study. Scand J Urol Nephrol.

[39] Kianbakht S, Abasi B, Dabaghian F (2013). Anti-hyperglycemic effect of vaccinium arctostaphylos in type 2 diabetic patients: a randomized controlled trial. Forschende Komplementärmedizin/Research in Complementary Medicine.

[40] Mahluji S, Attari VE, Mobasseri M, Payahoo L, Ostadrahimi A, Golzari SE (2013). Effects of ginger (Zingiber officinale) on plasma glucose level, HbA1c and insulin sensitivity in type 2 diabetic patients. International journal of food sciences and nutrition.

[41] Mobaseri M, Aliasgarzadeh A, Bahrami A, Zargami N, Tabrizi A, Ave G (2012). Efficacy of the total extract of urtica dioica on the glucose utilization by the human muscle cells. J Clin Diagn Res.

[42] Nickavar B, Mosazadeh G (2009). Influence of three morus species extracts on α-amylase activity. Iranian Journal of Pharmaceutical Research.

[43] Mozaffari-Khosravi H, Jalali-Khanabadi BA, Afkhami-Ardekani M, Fatehi F (2009). Effects of sour tea (Hibiscus sabdariffa) on lipid profile and lipoproteins in patients with type II diabetes. J Altern Complement Med.

[44] Mozaffari-Khosravi H, Jalali-Khanabadi BA, Afkhami-Ardekani M, Fatehi F, Noori-Shadkam M (2009). The effects of sour tea (Hibiscus sabdariffa) on hypertension in patients with type II diabetes. J Hum Hypertens.

[45] Nickavar B, Amin G (2010). Bioassay-guided separation of an alpha-amylase inhibitor anthocyanin from Vaccinium arctostaphylos berries. Z Naturforsch C.

[46] Parsaeyan N (2012). The effect of coriander seed powder consumption on atherosclerotic and cardioprotective indices of type 2 diabetic patients 2. Research.

[47] Ramezani M, Azarabadi M, Fallah Huseini H, Abdi H, Baher G, Huseini M (2008). The effects of Silybum marianum (L.) Gaertn. seed extract on glycemic control in type II diabetic patient’s candidate for insulin therapy visiting endocrinology clinic in baqiyatallah hospital in the years of 2006. Journal of Medicinal Plants.

[48] Rashidi AA, Mirhashemi SM, Taghizadeh M, Sarkhail P (2013). Iranian medicinal plants for diabetes mellitus: a systematic review. Pak J Biol Sci.

[49] Salehi P, Asghari B, Esmaeili MA, Dehghan H, Ghazi I (2013). α-Glucosidase and α-amylase inhibitory effect and antioxidant activity of ten plant extracts traditionally used in Iran for diabetes. Journal of Medicinal Plants Research.

[50] Sheikhpour R, Yaghmaei P (2012). A survey on herbal medicines for hypoglycemia in diabetic patients. Iranian Journal of diabetes and obesity.

[51] Shojaii A, Dabaghian FH, Goushegir A, Fard MA (2011). Antidiabetic plants of Iran. Acta Med Iran.

[52] Shojaii A, Goushegir A, Dabaghian FH, Abdollahi M, Huseini HF (2011). Herbs and herbal preparations for glycemic control in diabetes mellitus (a systematic review). Journal of Medicinal Plants Research.

[53] Vosough-Ghanbari S, Rahimi R, Kharabaf S, Zeinali S, Mohammadirad A, Amini S (2010). Effects of Satureja khuzestanica on serum glucose, lipids and markers of oxidative stress in patients with type 2 diabetes mellitus: a double-blind randomized controlled trial. Evidence-Based Complementary and Alternative Medicine.

[54] Ziai SA, Larijani B, Akhoondzadeh S, Fakhrzadeh H, Dastpak A, Bandarian F (2005). Psyllium decreased serum glucose and glycosylated hemoglobin significantly in diabetic outpatients. J Ethnopharmacol.

[55] Hasani-Ranjbar S, Larijani B, Abdollahi M (2009). A systematic review of the potential herbal sources of future drugs effective in oxidant-related diseases. Inflammation and Allergy - Drug Targets.

[56] Hasani-Ranjbar S, Jouyandeh Z, Qorbani M, Hemmatabadi M, Larijani B (2012). The effect of semelil (angipars(R)) on bone resorption and bone formation markers in type 2 diabetic patients. DARU.

[57] Mehri A, Hasani-Ranjbar S, Larijani B, Abdollahi M (2011). A systematic review of efficacy and safety of Urtica dioica in the treatment of diabetes. Int J Pharmacol.

[58] Hasani-Ranjbar S, Nayebi N, Larijani B, Abdollahi M (2010). A systematic review of the efficacy and safety of Teucrium species; from anti-oxidant to anti-diabetic effects. Int J Pharmacol.

[59] Gholam Hosseinian A, Falah Hossein SF, Mirtaj Aldini S (2008). The inhibitory effect of some Iranian plants extracts on the alpha glucosidase. Iranian Journal of Basic Medical Sciences.

[60] Asgary S, Kelishadi R, Rafieian-Kopaei M, Najafi S, Najafi M, Sahebkar A (2013). Investigation of the lipid-modifying and antiinflammatory effects of cornus mas L. Supplementation on dyslipidemic children and adolescents. Pediatric cardiology.

[61] Asgary S, Naderi GH, Sarrafzadegan N, Mohammadifard N, Mostafavi S, Vakili R (2000). Antihypertensive and antihyperlipidemic effects of Achillea wilhelmsii. Drugs Exp Clin Res.

[62] Bathaei FS, Akhondzadeh S (2008). Cardiovascular effects of allium sativum (garlic): an evidence-based review. Journal of Tehran University Heart Center.

[63] Emtiazy M, Keshavarz M, Khodadoost M, Kamalinejad M, Gooshahgir SA, Shahrad Bajestani H (2012). Relation between body humors and hypercholesterolemia: an iranian traditional medicine perspective based on the teaching of avicenna. Iran Red Crescent Med J.

[64] Fallah Huseini H, Fakhrzadeh H, Dastpak A, Azarabadi M, Mohtashami TR (2005). Review of antihyperlipedemic herbal medicine. Journal of Medicinal Plants.

[65] Hasani-Ranjbar S, Nayebi N, Moradi L, Mehri A, Larijani B, Abdollahi M (2010). The efficacy and safety of herbal medicines used in the treatment of hyperlipidemia; a systematic review. Curr Pharm Des.

[66] Kianbakht S, Abasi B, Perham M, Hashem Dabaghian F (2011). Antihyperlipidemic effects of Salvia officinalis L. leaf extract in patients with hyperlipidemia: a randomized double-blind placebo-controlled clinical trial. Phytother Res.

[67] Kojuri J, Vosoughi AR, Akrami M (2007). Effects of anethum graveolens and garlic on lipid profile in hyperlipidemic patients. Lipids Health Dis.

[68] Mansouri M, Nayebi N, Hasani-Ranjbar S, Taheri E, Larijani B (2012). The effect of 12 weeks Anethum graveolens (dill) on metabolic markers in patients with metabolic syndrome; a randomized double blind controlled trial. DARU Journal of Pharmaceutical Sciences.

[69] Panahi Y, Pishgoo B, Beiraghdar F, Araghi ZM, Sahebkar A, Abolhasani E (2011). Results of a randomized, open-label, clinical trial investigating the effects of supplementation with Heracleum persicum extract as an adjunctive therapy for dyslipidemia. Scientific World Journal.

[70] Panahi Y, Pishgoo B, Jalalian HR, Mohammadi E, Taghipour HR, Sahebkar A (2012). Investigation of the effects of chlorella vulgaris as an adjunctive therapy for dyslipidemia: results of a randomised open‒label clinical trial. Nutrition & Dietetics.

[71] Sabzghabaee AM, Dianatkhah M, Sarrafzadegan N, Asgary S, Alireza G (2012). Clinical evaluation of Nigella sativa seeds for the treatment of hyperlipidemia: a randomized, placebo controlled clinical trial. Medicinski arhiv.

[72] Sahebkar A, Panahi Y, Jalalian HR, Pishgoo B, Mohammadi E, Abolhasani E (2011). Investigation of the effects of < i > chlorella vulgaris</i > as an adjunctive therapy for dyslipidemia: results of a randomized open-label clinical trial. Clin Biochem.

[73] Asghari M, Naseri M, Sabet Z, Davati A, Kamalinejad M, Jalali-Nadoushan MR (2013). Efficacy and safety of Ziabites (an Iranian traditional medicine compound) on glycemic control in type 2 diabetic patients. Journal of Medicinal Plants Research.

[74] Kianbakht S, Abasi B, Hashem Dabaghian F (2013). Improved lipid profile in hyperlipidemic patients taking vaccinium arctostaphylos fruit hydroalcoholic extract: a randomized double-blind placebo-controlled clinical trial. Phytother Res.

[75] Asghari SD (2012). Pharmacognostic evaluation of herbal medicines used for obesity on Isfahan traditional medicine market. Research in Pharmaceutical Sciences.

[76] Atashak S, Peeri M, Azarbayjani MA, Stannard SR, Haghighi MM (2011). Obesity-related cardiovascular risk factors after long- term resistance training and ginger supplementation. Journal of Sports Science and Medicine.

[77] Atashak S, Peeri M, Jafari A, Azarbayjani MA (2011). Effects of ginger supplementation and resistance training on lipid profiles and body composition in obese men. Journal of Medicinal Plants Research.

[78] Hasani-Ranjbar S, Jouyandeh Z, Abdollahi M (2013). A systematic review of anti-obesity medicinal plants - an update. J Diabetes Metab Disord.

[79] Hasani-Ranjbar S, Nayebi N, Larijani B, Abdollahi M (2009). A systematic review of the efficacy and safety of herbal medicines used in the treatment of obesity. World J Gastroenterol.

[80] Hashemipoor M, Kelishadi R, Asgary Sedigheh MFN, Tavakkoli N (2003). Efficacy of two different herbal medical therapy in controlling childhood obesity. Journal of Research in Medical Sciences (Jrms).

[81] Khazan M, Hedayati M, Askari S, Azizi F (2013). Adulteration of products sold as Chinese herbal medicines for weight loss with thyroid hormones and PCP. Journal of Herbal Medicine.

[82] Mirzaei K, Hossein-nezhad A, Aslani S, Emamgholipour S, Karimi M, Keshavarz SA (2012). Energy expenditure regulation via macrophage migration inhibitory factor in obesity and in vitro anti-macrophage migration inhibitory factor effect of Alpinia officinarum hance extraction. Endocr Pract.

[83] Moghaddasi MS, Kashani HH (2012). Effect of tea in the treatment of obesity. Scientific Research and Essays.

[84] Sabzghabaee AM, Ataei E, Kelishadi R, Ghannadi A, Soltani R, Badri S (2013). Effect of hibiscus sabdariffa calices on dyslipidemia in obese adolescents: a triple-masked randomized controlled trial. atherosclerosis.

[85] Zare H, Sarvestani F (2012). Combined effects of physical exercise and green tea on obese people. International Journal of Ayurvedic and Herbal Medicine.

[86] Mohammad K, Larijani B (2013). A systematic review of the antioxidant, anti-diabetic, and anti-obesity effects and safety of triphala herbal formulation. Journal of Medicinal Plants Research.

[87] Kamali SH, Khalaj AR, Hasani-Ranjbar S, Esfehani MM, Kamalinejad M, Larijani B (2013). A systematic review of the antioxidant, anti-diabetic, and anti-obesity effects and safety of triphala herbal formulation. Journal of Medicinal Plants Research.

[88] Azadbakht M, Baheddini A, Shorideh SM, Naserzadeh A (2005). Leaf and fruit flavonoidal extracts on serum prolactin concentration. Effect of Vitex agnus - Castus L. Journal of Medicinal Plants.

[89] Hasani-Ranjbar S, Vahidi H, Taslimi S, Karimi N, Larijani B, Abdollahi M (2010). A systematic review on the efficacy of herbal medicines in the management of human drug-induced hyperprolactinemia: potential sources for the development of novel drugs. Int J Pharmacol.

[90] Asgarpanah J, Haghighat E: **An overview on phytochemistry and pharmacologic properties of rhus coriaria L.** *Journal of Pharmaceutical & Health Sciences* 2012.,**1**(4):

[91] Bahramikia S, Yazdanparast R (2012). Phytochemistry and medicinal properties of Teucrium polium L. (Lamiaceae). Phytother Res.

[92] Iranshahy M, Iranshahi M (2011). Traditional uses, phytochemistry and pharmacology of asafoetida (Ferula assa-foetida oleo-gum-resin)-a review. J Ethnopharmacol.

[93] Mikaili P, Shayegh J, Asghari MH, Sarahroodi S, Sharifi M (2011). Currently used traditional phytomedicines with hot nature in Iran. Annals of Biological Research Annals of Biological Research.

[94] Mirdeilami SZ, Barani H, Mazandarani M, Heshmati GA (2011). Ethnopharmacological survey of Medicinal Plants in Maraveh Tappeh Region, North of Iran. Iranian Journal of Plant Physiology.

[95] Mombeini T, Mombeini M, Aghayi M (2009). Evaluation of pharmacological effects of Origanum genus (Origanum spp.). Journal of Medicinal Plants.

[96] Mosaddegh M, Naghibi F, Moazzeni H, Pirani A, Esmaeili S (2012). Ethnobotanical survey of herbal remedies traditionally used in Kohghiluyeh va Boyer Ahmad province of Iran. Journal of ethnopharmacology.

[97] Rahimi R, Amin G, Ardekani MRS (2012). A review on citrullus colocynthis schrad: from traditional iranian medicine to modern phytotherapy. The Journal of Alternative and Complementary Medicine.

[98] Rahimi R, Ardekani MRS (2013). Medicinal properties of Foeniculum vulgare Mill. in traditional Iranian medicine and modern phytotherapy. Chinese journal of integrative medicine.

[99] Shojaii A, Abdollahi Fard M (2012). Review of pharmacological properties and chemical constituents of pimpinella anisum. ISRN Pharm.

[100] Zare AR, Omidi M, Fallah Hoseini H, Yazdani D, Sh R, Irvani N (2011). A review on pharmacological effects of Ferula assa - Foetida L. A systematic review. Journal of Medicinal Plants.

