# Supplementary figures and images for: Trends in publication of evidence-based Traditional Iranian medicine in endocrinology and metabolic disorders
Source: J Diabetes Metab Disord. 2013 Dec 19;12:49. doi: 10.1186/2251-6581-12-49 (PMC7968339; doi:10.1186/2251-6581-12-49)

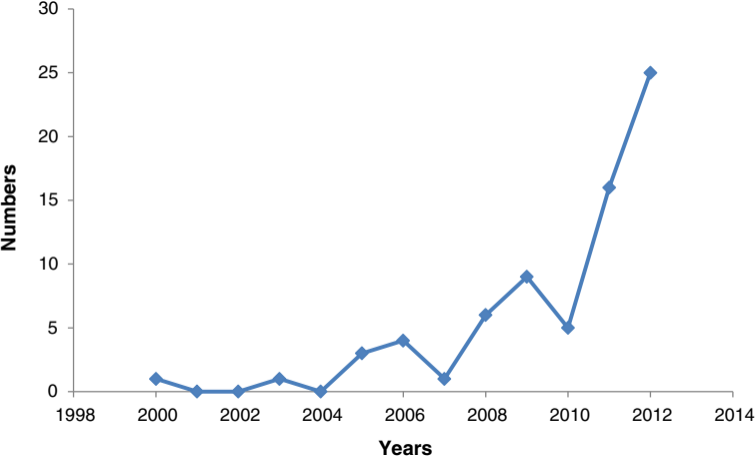

Supplement: Supplementary file 1 — Authors’ original file for figure 1 [file 40200_2013_181_MOESM1_ESM.pdf]
